# Preferential inhibition of xanthine oxidase by 2-amino-6-hydroxy-8-mercaptopurine and 2-amino-6-purine thiol

**DOI:** 10.1186/1471-2091-8-8

**Published:** 2007-05-18

**Authors:** Sukirti Kalra, Gopabandhu Jena, Kulbhushan Tikoo, Anup Kumar Mukhopadhyay

**Affiliations:** 1 Department of Biotechnology, National Institute of Pharmaceutical Education and Research (NIPER), Sector 67, Phase X, S.A.S Nagar, Mohali, Punjab,160062 India; 2 Department of Pharmacology and Toxicology, National Institute of Pharmaceutical Education and Research (NIPER), Sector 67, Phase X, S.A.S Nagar, Mohali, Punjab, 160062 India; 3 Department of Biotechnology, National Institute of Pharmaceutical Education and Research (NIPER), Sector 67, Phase X, S.A.S Nagar, Mohali, Punjab, 160062 India

## Abstract

**Background:**

The anticancer drug, 6-mercaptopurine (6MP) is subjected to metabolic clearance through xanthine oxidase (XOD) mediated hydroxylation, producing 6-thiouric acid (6TUA), which is excreted in urine. This reduces the effective amount of drug available for therapeutic efficacy. Co-administration of allopurinol, a suicide inhibitor of XOD, which blocks the hydroxylation of 6MP inadvertently enhances the 6MP blood level, counters this reduction. However, allopurinol also blocks the hydroxylation of hypoxanthine, xanthine (released from dead cancer cells) leading to their accumulation in the body causing biochemical complications such as xanthine nephropathy. This necessitates the use of a preferential XOD inhibitor that selectively inhibits 6MP transformation, but leaves xanthine metabolism unaffected.

**Results:**

Here, we have characterized two such unique inhibitors namely, 2-amino-6-hydroxy-8-mercaptopurine (AHMP) and 2-amino-6-purinethiol (APT) on the basis of IC_50 _values, residual activity in bi-substrate simulative reaction and the kinetic parameters like *K*_m_, *K*_i_, *k*_cat_. The IC_50 _values of AHMP for xanthine and 6MP as substrate are 17.71 ± 0.29 μM and 0.54 ± 0.01 μM, respectively and the IC_50 _values of APT for xanthine and 6MP as substrates are 16.38 ± 0.21 μM and 2.57 ± 0.08 μM, respectively. The *K*_i _values of XOD using AHMP as inhibitor with xanthine and 6MP as substrate are 5.78 ± 0.48 μM and 0.96 ± 0.01 μM, respectively. The *K*i values of XOD using APT as inhibitor with xanthine and 6MP as substrate are 6.61 ± 0.28 μM and 1.30 ± 0.09 μM. The corresponding *K*_m _values of XOD using xanthine and 6MP as substrate are 2.65 ± 0.02 μM and 6.01 ± 0.03 μM, respectively. The results suggest that the efficiency of substrate binding to XOD and its subsequent catalytic hydroxylation is much superior for xanthine in comparison to 6MP. In addition, the efficiency of the inhibitor binding to XOD is much more superior when 6MP is the substrate instead of xanthine. We further undertook the toxicological evaluation of these inhibitors in a single dose acute toxicity study in mice and our preliminary experimental results suggested that the inhibitors were equally non-toxic in the tested doses.

**Conclusion:**

We conclude that administration of either APT or AHMP along with the major anti-leukemic drug 6MP might serve as a good combination cancer chemotherapy regimen.

## Background

**Figure 1:**
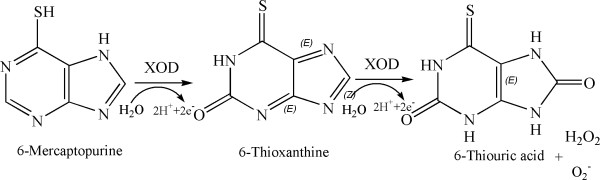
Scheme showing oxidative hydroxylation of 6-mercaptopurine to 6-thioxanthine to 6-thiouric acid.

6MP, an analog of hypoxanthine was first among the thiopurine series found to be useful as an anticancer drug to treat ALL, the most common malignancy affecting children and other leukemias [1,2]. 6MP is taken up by the cell and is transformed into an active metabolite 6-thio-inosine monophosphate, an inhibitor of DNA synthesis, by the target enzyme hypoxanthine-guanine phosphoribosyltransferase (HGPRT) using phosphoribosyl-pyrophosphate (PRPP) as a co-substrate [2,3]. 6MP is also inadvertently being utilized by another enzyme XOD leading to the generation of an inactive metabolite, 6TUA which is excreted in urine [4,5]. Levels of XOD expression varies from tissue to tissue and is also known to be over-expressed in tumors [6-10]. So, in such cases, when the anticancer drug 6MP is administered, it would possibly lead to more metabolic transformation of 6MP. XOD catalyzes a two-step hydroxylation reaction of 6MP, leading to formation of 8-OH-6MP first and subsequently to 6TUA (Figure 1).

The wasteful degradation of 6MP by XOD suggested that it is highly essential to minimize this catabolic pathway by the use of a XOD inhibitor. Previous reports indicated that semicarbazide could inhibit XOD and XDH activities *in vitro *as well as *in vivo *but the inhibition *in vivo *was less than 50% at doses that produced significant toxic effects [6].

**Figure 2:**
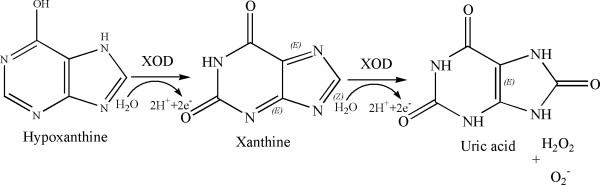
Scheme showing oxidative hydroxylation of hypoxanthine to xanthine to uric acid.

Then, allopurinol, a pyrazolopyrimidine derivative and an analog of hypoxanthine, was employed as a part of combination cancer therapy along with 6MP and was found to result in a noticeable drop in the pace of catabolism of 6-substituted purines including 6MP as well as potentiate the antitumor and immunosuppressive properties of 6MP upto three to four-folds [6,11,12]. Allopurinol is a non-specific suicide inhibitor of XOD available in market for the treatment of gout, caused by the accumulation of uric acid crystals in the joints and tissues [13-15]. XOD is an important purine metabolism pathway enzyme which catalyzes the oxidative hydroxylation of the natural purine, hypoxanthine to xanthine to finally uric acid which is excreted in the urine [16-18] (Figure 2). Allopurinol usage in the combination chemotherapy with 6MP increases plasma concentration of the anticancer drug allowing the reduction in the large 6MP dose to almost 25% [19-21]. On the other hand, allopurinol displays certain biochemical complications; the most important amongst these is leading to the accumulation of natural purines hypoxanthine and xanthine, as along with 6MP, allopurinol also inhibits the natural hydroxylation pathway of hypoxanthine and xanthine to uric acid. This accumulation of natural purines leads to xanthine nephropathy [22]. Furthermore, a huge amount of reactive oxygen species (ROS) is generated with the administration of allopurinol [23].

So, alternatively a substrate specific inhibitor along with administration of this multipotential drug, 6MP must be used. Diphenyleneiodonium chloride has been reported as the preferential inhibitor of NAD(P)H oxidase as it inhibits the NADH oxidation more effectively than of NADPH [24]. Human PC4 is also been reported as a substrate specific inhibitor of the RNA Polymerase II phosphorylation [25]. Inspite of the humungous progress towards the development of XOD inhibitors to date [6,11,26-30], there has been no report of any such substrate specific inhibitor that selectively prevents 6MP from being wastefully transformed into 6TUA against natural purines. In the present work, we have discussed the action of two such preferential XOD inhibitors.

## Results

A preferential XOD inhibitor is the one which will inhibit 6MP from being metabolized by the action of XOD while enzymatic hydroxylation of xanthine to uric acid is continued. The hydroxylation reaction of hypoxanthine forming uric acid and 6MP forming 6TUA has no common intermediates (Figures 1 and 2).

### IC_50 _determination

A few purine and pyrazolo pyrimidine-based compounds were screened for inhibitory property against XOD-catalyzed hydroxylation using xanthine and 6MP as substrates (data not shown). 10 μM of either of the substrates and 2.8 U/ml of XOD were taken for the IC_50 _determination (Table 1). IC_50 _values of allopurinol for the enzymatic conversion of xanthine to uric acid and 6MP to 6TUA are found to be almost same (2.36 ± 0.03 and 1.92 ± 0.03 μM) and therefore, suggest that allopurinol is equally efficient in inhibiting enzymatic conversion of both xanthine to uric acid and 6MP to 6TUA formation. Since allopurinol is a suicide inhibitor of XOD and the inhibition develops time-dependently [31], so the IC_50 _value of allopurinol was calculated for the late phase as well by carrying out the reaction for 60 min and the IC_50 _values of allopurinol for XOD corresponding to the substrates hypoxanthine and xanthine were 0.55 ± 0.45 and 1.65 ± 0.25 μM, respectively. While for 6MP, the IC_50 _values were already calculated from 60 min reaction. Among many compounds tested, two purine-based compounds namely, AHMP and APT demonstrated a lower IC_50 _value for the 6MP to 6TUA reaction (0.54 ± 0.01 μM and 2.57 ± 0.08 μM, respectively), although showed a reasonably higher IC_50 _value for the xanthine to uric acid formation reaction (17.71 ± 0.29 μM and 16.38 ± 0.21 μM, respectively). The lower IC_50 _values for 6MP hydroxylation than xanthine indicate that AHMP and APT inhibit hydroxylation of 6MP and xanthine with variable efficiencies. These two purine-based compounds are found to be showing no such difference in the IC_50 _value in the first and the late phases and none were found to be XOD substrates. However, like allopurinol, the recently discovered XOD inhibitors, 6-(N-benzoylamino)purine [29] and also 4-aminopyrazolo 3, 4-d pyrimidine (APP) [30] did not exhibit any preferential inhibition of the 6MP hydroxylation over xanthine. The results imply that the structure of the inhibitor plays a major role in regulating this preferential inhibition mechanism.

**Table 1:** Table showing IC_50 _values of different purine and allopurinol-based compounds for XOD inhibition.

		**(μM ± SEM)**		
		
**Inhibitor**		**Hypoxanthine**	**Xanthine**	**6MP**
ALLOPURINOL		0.91 ± 0.01	2.36 ± 0.03	1.92 ± 0.03
APT		7.28 ± 0.17	16.38 ± 0.21	2.57 ± 0.08
AHMP		5.08 ± 0.04	17.71 ± 0.29	0.54 ± 0.01
APP		6.79 ± 0.28	20.76 ± 0.84	12.86 ± 0.69

The reaction mixture comprised of 1 ml of 0.2 M, sodium phosphate buffer pH 7.4 containing 10 μM of one of the substrates namely, hypoxanthine, xanthine or 6MP. The concentration range of allopurinol and APP were 0–3 μM and 0–100 μM, respectively for all three substrates. The concentration range of AHMP and APT were 0–100 μM and 0–50 μM, respectively for all three substrates. The reaction was initiated by the addition of 2.8 U/ml of bovine XOD.

We also monitored the substrate-induced enzymatic electron transfer to the acceptors like 2,6-dichlorophenol indophenol (DCPIP) and cytochrome c and found that these two preferential inhibitors did not release either any electrons or generate any ROS on interaction with XOD while allopurinol led to substrate-induced enzymatic electron transfer via DCPIP (data not shown) [29,30]. Some researchers report the generation of superoxides from allopurinol in large amounts [23].

### *In vitro *bi-substrate-inhibitor-enzyme simulation

In order to determine the extent of enzymatic hydroxylation of xanthine and 6MP into their corresponding products when the two substrates were present together in the same reaction mixture, we carried out a bi-substrate simulative experiment.

**Figure 3:**
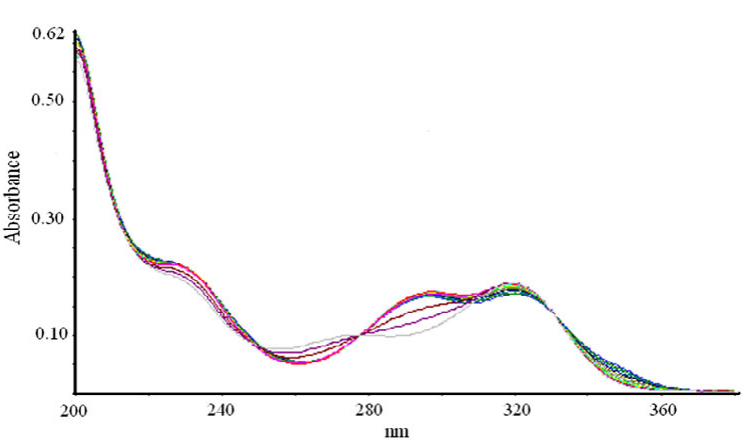
**Bi-substrate reaction of XOD-mediated hydroxylation of xanthine and 6MP**. The reaction mixture comprised of 1 ml of 0.2 M, sodium phosphate buffer pH 7.4 containing 10 μM of both xanthine and 6MP. The reaction was initiated by the addition of 2.8 U/ml of bovine XOD and observed for 20 min.

**Figure 4:**
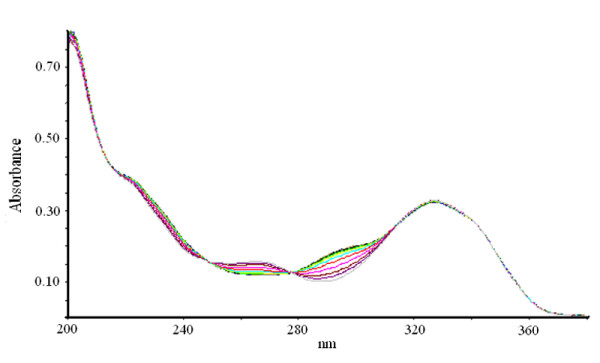
**Bi-substrate reaction of XOD-mediated hydroxylation of xanthine and 6MP in presence of APT**. The reaction mixture comprised of 1 ml of 0.2 M, sodium phosphate buffer pH 7.4 containing 10 μM of both xanthine and 6MP. 5 μM APT was taken. The reaction was initiated by the addition of 2.8 U/ml of bovine XOD and observed for 20 min.

**Figure 5:**
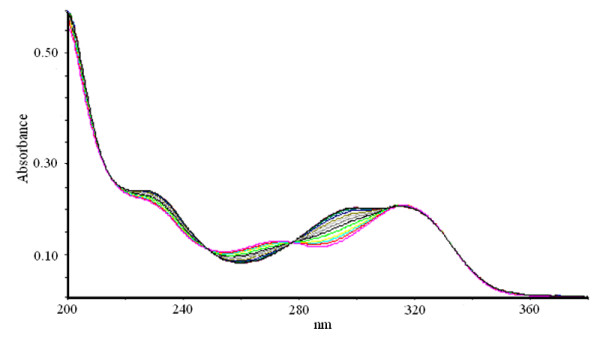
**Bi-substrate reaction of XOD-mediated hydroxylation of xanthine and 6MP in presence of AHMP**. The reaction mixture comprised of 1 ml of 0.2 M, sodium phosphate buffer pH 7.4 containing 10 μM of both xanthine and 6MP. 2 μM AHMP was taken. The reaction was initiated by the addition of 2.8 U/ml of bovine XOD and observed for 20 min.

Percent residual activity for generation of uric acid and 6TUA was determined in the presence of various concentrations of AHMP, APT and allopurinol (Table 2). XOD-mediated hydroxylation reaction of xanthine and 6MP (bi-substrate) led to generation of uric acid and 6TUA with progress of time, as shown in Figure 3. Absorption spectra of the time dependent XOD-mediated bi-substrate reaction in the presence of APT and AHMP are depicted in Figure 4 and Figure 5, respectively. Almost complete inhibition of 6MP hydroxylation while little inhibition of the xanthine hydroxylation reaction was achieved in the presence of 5 μM APT and 2 μM AHMP (Here, variable inhibitor concentrations were chosen depending on different IC_50 _values for the two substrates). Accounting for the percentage inhibition using these inhibitors, we found that using 2.5 μM allopurinol, the uric acid and 6TUA forming residual enzyme activity was 30% and 16%, respectively. Using 2.5 μM APT, the residual enzyme activity for xanthine and 6MP hydroxylation was reduced to 80% and 25%, respectively and while using 2.5 μM AHMP it resulted in residual 64% uric acid formation activity and 18% 6TUA formation.

**Table 2:** Bi-substrate simulative experiment.

**Inhibitor (μM)**	**% Activity with xanthine as substrate**	**% Activity with 6MP as substrate**
ALLOPURINOL 0.5 1.0 2.5	95 ± 1.09 68 ± 3.51 30 ± 1.04	83 ± 1.05 48 ± 1.07 16 ± 1.05
APT 0.5 1.0 2.5	96 ± 1.00 90 ± 0.51 80 ± 2.50	73 ± 2.50 66 ± 3.01 25 ± 2.06
AHMP 0.5 1.0 2.5	90 ± 1.09 85 ± 2.06 64 ± 1.02	59 ± 4.01 47 ± 4.01 18 ± 2.08

The reaction mixture comprised of 1 ml of 0.2 M, sodium phosphate buffer pH 7.4 containing 10 μM of both xanthine and 6MP. The concentration range of allopurinol was 0–2.5 μM. The concentration range of AHMP was 0–3 μM. The concentration range of APT was 0–10 μM. The reaction was initiated by the addition of 2.8 U/ml of bovine XOD.

Therefore, the results suggest that when the two substrates, 6MP and xanthine are present together along with either of the preferential inhibitors, the conversion of xanthine to uric acid is more favored than 6MP to 6TUA conversion. The results also suggest that a critical concentration of the inhibitor could be used to effectively inhibit the enzymatic conversion of 6MP to 6TUA in the branched pathway. A similar *invitro *tri-substrate simulative studies was carried out with three substrates of XOD namely, hypoxanthine, xanthine and 6MP in the absence and presence of preferential inhibitors. Identical results corresponding to the preferential inhibitors were obtained indicating the discriminatory inhibition of the 6MP to 6TUA conversion and not hypoxanthine to uric acid conversion by the action of AHMP and APT (data not shown).

### Mechanism of inhibition of Xanthine oxidase by AHMP and APT

**Figure 6:**
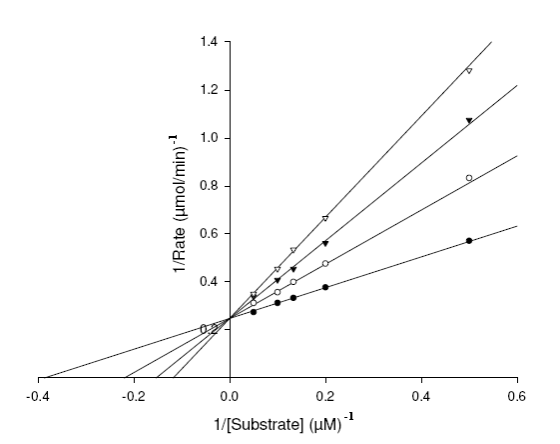
**Lineweaver-Burk plot of inhibition of XOD-mediated xanthine hydroxylation by APT**. The reaction mixture comprised of 1 ml of 0.2 M, sodium phosphate buffer pH 7.4 containing varying concentrations of xanthine ranging from 2, 5, 8, 10 and 20 μM. APT concentrations were [I] = 0 μM (●), [I] = 5 μM (○), [I] = 10 μM (▼), [I] = 15 μM (▽). The reaction was initiated by the addition of 2.8 U/ml of bovine XOD.

**Figure 7:**
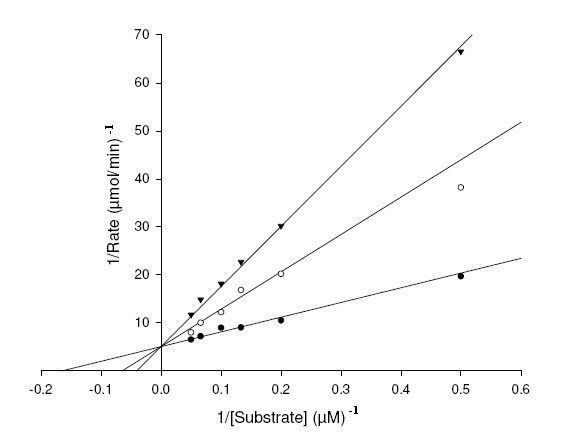
**Lineweaver-Burk plot of inhibition of XOD-mediated 6MP hydroxylation by APT**. The reaction mixture comprised of 1 ml of 0.2 M, sodium phosphate buffer pH 7.4 containing varying concentrations of 6MP ranging from 2, 5, 8, 10, 15 and 20 μM. APT concentrations were [I] = 0 μM (●), [I] = 2 μM (○), [I] = 4 μM (▼). The reaction was initiated by the addition of 2.8 U/ml of bovine XOD.

**Figure 8:**
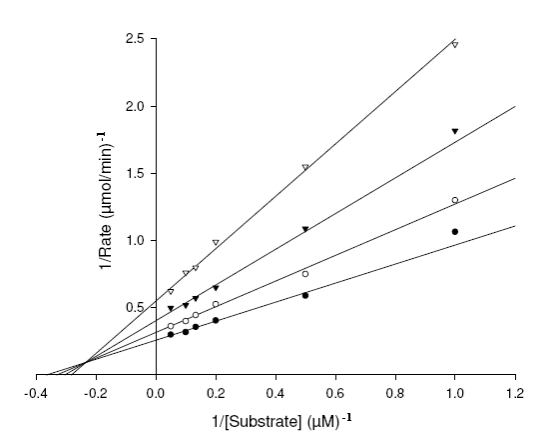
**Lineweaver-Burk plot of inhibition of XOD-mediated xanthine hydroxylation by AHMP**. The reaction mixture comprised of 1 ml of 0.2 M, sodium phosphate buffer pH 7.4 containing varying concentrations of xanthine ranging from 1, 2, 5, 8, 10 and 20 μM. AHMP concentrations were [I] = 0 μM(●), [I] = 2 μM (○), [I] = 5 (▼), [I] = 10 μM (▽). The reaction was initiated by the addition of 2.8 U/ml of bovine XOD.

**Figure 9:**
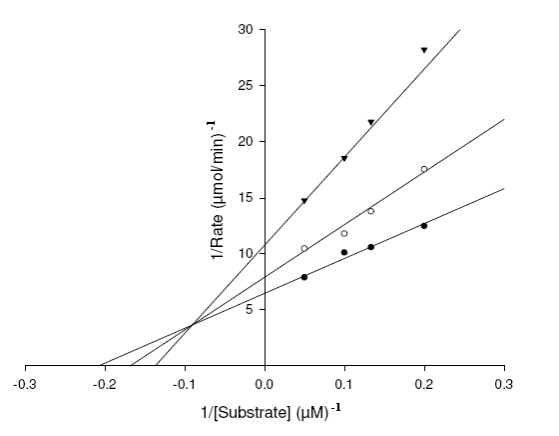
**Lineweaver-Burk plot of inhibition of XOD-mediated 6MP hydroxylation by AHMP**. The reaction mixture comprised of 1 ml of 0.2 M, sodium phosphate buffer pH 7.4 containing varying concentrations of 6MP ranging from 5, 8, 10 and 20 μM. AHMP concentrations were [I] = 0 μM (●), [I] = 0.5 μM (○), [I] = 1.5 μM (▼). The reaction was initiated by the addition of 2.8 U/ml of bovine XOD.

To study mechanism of inhibition and distinguish the interaction of XOD with the substrates, xanthine and 6MP and inhibitors, AHMP and APT; we studied the steady-state kinetics of XOD with respect to the substrates and inhibitors. The double reciprocal Lineweaver Burk (LB) plots were drawn to find the mechanism of inhibition of XOD by APT and AHMP (Figure 6, 7, 8, 9). The *K*_m_, *K*_i_^1^, *K*_I_^1^, *K*_i_^2^, *K*_I_^2 ^and *k*_cat _values, and inhibitory mechanism exhibited by APT and AHMP are all depicted in Table 3.

**Table 3:** Table showing kinetic parameters of APT and AHMP.

**Inhibitor**	**Substrate**	***K*_m _(μM)**	**Mechanism of inhibition**	***K*_i _(μM)**	***k*_cat_(min)**^-1^
APT	Xanthine	2.65 ± 0.02	Competitive	6.61 ± 0.28	114.54 ± 0.24
	6MP	6.01 ± 0.03	Competitive	1.30 ± 0.09	2.85 ± 0.02
AHMP	Xanthine	2.71 ± 0.05	Mixed	5.7 ± 0.48(*K*_i_^2^) (Competitive) 6.24 ± 0.04 (*K*_I_^2^) (Non-competitive)	128.57 ± 0.45
	6MP	4.89 ± 0.04	Mixed	0.96 ± 0.01(*K*_i_^2^) (Competitive) 0.98 ± 0.06 (*K*_I_^2^) (Non-competitive)	3.64 ± 0.04

The reaction mixture comprised of 1 ml of 0.2 M, sodium phosphate buffer pH 7.4 containing 10 μM of either xanthine or 6MP. The reaction was initiated by the addition of 2.8 U/ml of bovine XOD.

*K*_i_^2 ^= Inhibition constant for competitive inhibition mechanism exhibited by AHMP

*K*_I_^2 ^= Inhibition constant for non-competitive inhibition mechanism exhibited by AHMP

Figure 6 depicts the LB plot of the steady-state inhibition with increasing APT concentrations, which show straight lines meeting at a point on Y-axis. Mechanistically, this type of inhibition is interpreted as competitive. Structurally speaking, we observe that the 8^th ^position in both xanthine and APT is free for hydroxylation facing the Mo site of XOD. Also, both APT and xanthine has substitution at the same positions, i.e. 2^nd ^and 6^th^. Therefore, it seems that xanthine and APT compete for XOD active site. Consequently, it becomes evident that APT will bind to XOD at its substrate binding site. The *K*_i_^1 ^value estimated from the LB plot using the equation (1) [mentioned in Methods section] were equivalent to 6.6 ± 0.28 μM with respect to xanthine (Table 3).

Figure 7 projects a LB plot of steady-state inhibition of XOD using 6MP as substrate with increasing APT concentrations, suggesting a conventional competitive inhibition mechanism. Structural explanations illustrate that 8^th ^position is open for action in both 6MP and APT in front of the Mo site of XOD. The *K*_i_^1 ^value determined from the LB plot using the equation (1) [mentioned in Methods section] was found to be 1.30 ± 0.09 μM (Table 3).

AHMP also showed signs of efficiently inhibiting the XOD-catalyzed 6MP catabolic reaction than the xanthine hydroxylation reaction. Figure 8 demonstrates the LB plot of the steady-state inhibition of XOD using xanthine as substrate with increasing AHMP concentrations, illustrating the mixed type of inhibition like known XOD inhibitors, BOF-4272, Pd^+2^, Y-700, Morin and Galagin, [32-35]. The *K*_i_^2 ^and *K*_I_^2 ^values of 5.78 ± 0.48 μM (competitive) and 6.24 ± 0.04 μM (non-competitive) were calculated from the LB plot using the equation (4) and (5) [mentioned in Methods section]. Its non-competitive inhibition property can be explained on the basis that 8^th ^position is occupied by SH group in AHMP, which is found free in xanthine. Besides, the presence of NH_2 _group at 2^nd ^position in AHMP in place of an OH group in xanthine makes it faultless contender to bind at any site other than active site on XOD. And its competitive inhibition property may be due to the presence of OH group present at the 6^th ^position in both AHMP and xanthine and thus, they compete for the enzyme active site.

For XOD-catalyzed 6MP catabolic reaction, AHMP shows evidence of a mixed inhibition mechanism (Figure 9) indicating that native XOD is capable of forming two inhibitory complexes with AHMP. Occurrence of SH group at positions 6^th ^in 6MP and at 8^th ^in AHMP makes the latter an ideal applicant to compete with 6MP for active site and consequent inhibition of XOD. However, the existence of extra groups (OH and NH_2_) in AHMP at two positions makes it attach at a site other than the active site of the enzyme. *K*_i_^2 ^and *K*_I_^2 ^values for inhibition were calculated out to be 0.96 ± 0.01 μM (competitive) and 0.98 ± 0.06 μM (non-competitive) using the equations (4) and (5) [mentioned in Methods section]. The values of the two inhibition constants of AHMP do not differ much, indicating that the nature of the coordination of the AHMP for the competitive and non-competitive binding sites is similar.

### Comparison and evaluation of acute toxicity

Single dose acute toxicity was evaluated for AHMP and APT and compared with allopurinol, the standard drug used in the present investigation. No differences in general behavioral toxicity and no mortality were observed after the administration of the compounds. No significant changes in the initial and final body weight were observed for any of the compounds. The weekly food intake decreased for the groups treated with AHMP, APT and allopurinol as compared to vehicle treated groups. However, AHMP and APT treated groups food intake was marginally less as compared to allopurinol treated groups. Gross necropsy revealed no changes in vital organs. No difference in organ weight was observed as compared with the control groups.

## Discussion

### Inhibition mechanism of Xanthine oxidase by AHMP and APT

Mixed-type inhibition includes competitive and non-competitive inhibition. Competitive inhibition is exhibited when the substrate and the inhibitor compete for the substrate binding active site but non-competitive inhibition is exhibited when the inhibitor binds at a site other than the active site. Following the crystallographic studies performed on XOD, no peripheral binding site has been reported in XOD catalytic subunits [36,37]. For the past 50 years, it was assumed that the two XOD subunits carry out catalysis independently but a recent report [38] on the cooperative binding of the two subunits of XOD may support the non-competitive inhibition mechanism exhibited by AHMP. AHMP possibly interacts with the XOD domains distal to the substrate binding site also other than the substrate binding site, thereby possibly resulting in allosteric effects that attenuate the enzymatic activity.

Since XOD acts via a ping-pong mechanism, alternating between the oxidized and reduced forms [39], so another plausible explanation for the mixed inhibition exhibited by AHMP is that AHMP binds to both the oxidized and reduced form of XOD. Allopurinol (a known weak competitive inhibitor) and nitric oxide are known to strongly bind to the reduced state of XOD [40,41]. APT being a competitive inhibitor thus, might not be binding to the oxidized state but to the reduced state of XOD. The difference in the mechanism of inhibition exhibited by AHMP and APT must be possible due to the structural dissimilarities between the two inhibitors.

### Biochemical explanations of the efficiency of binding of substrate and inhibitor to Xanthine oxidase

The *K*_m_, *k*_cat_and *K*_i _values of XOD corresponding to the substrates, 6MP and xanthine and the inhibitors, APT and AHMP behave as the factors indicating the type of their binding and catalytic interactions with the enzyme. In the biochemical reaction,



where, E is enzyme, S is substrate, P is product, k_1 _and k_3 _are the rate constants for the forward reactions and k_2 _is the rate constant for the backward reaction.

By definition, *K*_m _of any biochemical reaction can be mathematically expressed in terms of rate constant [42] as

*K*_m _= (k_2_+ k_3_)/k_1_

Lower *K*_m _implies a lower dissociation of ES into E+S or E+P while higher *K*_m _implies higher dissociation of ES into E+S or E+P. While looking at the turnover number (*k*_cat_) of xanthine and 6MP, we found that it is the dissociation of the corresponding ES complexes into E+P and not E+S.

The inhibition constant *K*_i _on the other hand can be defined [42] as:



where, I is inhibitor, k_1 _is the rate constant for the forward reaction and k_2 _is the rate constant for the backward reaction

*K*_i _= k_2_/k_1_

Lower *K*_i _implies lower dissociation of EI complex into E + I and higher association of E and I, while higher *K*_i _means higher dissociation of EI complex and lower association of E and I.

*K*_m _and *k*_cat _values of XOD using 6MP as substrate implied that the conversion of XOD-6MP (ES) complex into its product 6TUA is slower as its turnover number is much lower (2.85 ± 0.02 μM/min) than the conversion of XOD-xanthine complex into its corresponding product (Table 3). Additionally, XOD-6MP (ES) complex is also less favored as the *K*_m _in this case is high (6.01 ± 0.03 μM). Considering xanthine as substrate of XOD however, the situation is different. The high *k*_cat _(114.54 ± 0.24 μM/min) indicates that the forward reaction of xanthine-XOD into uric acid (ES into E+P) is faster as compared to its dissociation into other counterpart (E+S) and lower *K*_m _(2.65 ± 0.02 μM) indicates that xanthine-XOD (ES) complex formation is also more favored. Therefore, the higher turnover of xanthine to uric acid conversion in comparison to 6MP to 6TUA conversion is favored only if the ES complex formation is favored rather than its dissociation into E+S which does not occur in case of 6MP-XOD interaction.

Now, we can presume a correlationship of *K*_m _and *K*_i _of XOD with respect to xanthine and 6MP, respectively. When 6MP and APT (or AHMP) interact with XOD, lower *K*_i _implies stronger XOD-AHMP (EI) complex formation and AHMP will more efficiently dissociate 6MP from XOD active site while higher *K*_m _implies less favored XOD-6MP (ES) complex formation and subsequently, slower 6TUA formation, further supporting that XOD and 6MP (E+S) formation is much preferred in 6MP as compared to xanthine. However, when xanthine and APT (or AHMP) interact with XOD, higher *K*_i _implies weaker and slower rate of formation of XOD-AHMP (EI) complex and the inhibitor AHMP is not efficient in displacing xanthine from XOD and thus, the enzymatic hydroxylation of xanthine continues while lower *K*_m _implies more favored formation of XOD-xanthine (ES) complex and subsequently, higher formation of uric acid.

**Figure 10:**
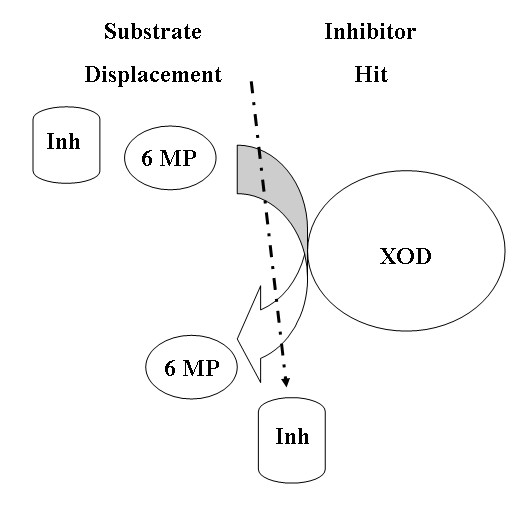
6-mercaptopurine-XOD-Inhibitor interaction showing inhibitor hit and substrate displacement.

**Figure 11:**
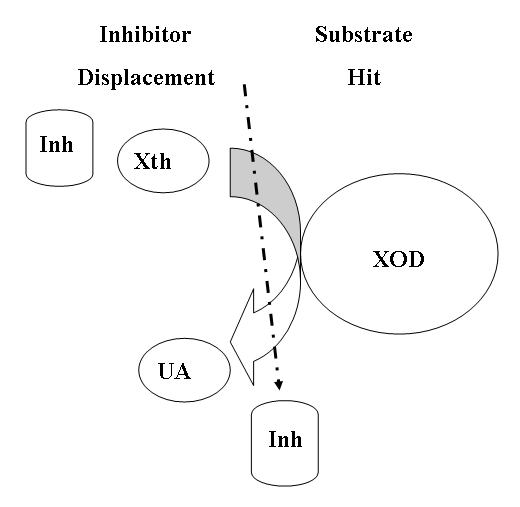
Xanthine-XOD-Inhibitor interaction showing substrate hit and inhibitor displacement.

It will be right to say that these preferential inhibitors, AHMP and APT displace 6MP efficiently from weak XOD-6MP complex while AHMP and APT are incapable of displacing xanthine from strong xanthine-XOD complex (Figures 10 and 11). In other words, substrate hit and inhibitor displacement takes place from XOD active or non-active site for xanthine-XOD reaction but inhibitor hit and substrate displacement for 6MP-XOD reaction. It can be stated that the 6MP to 6TUA hydroxylation is discriminately inhibited in contrast to xanthine to uric acid hydroxylation by these preferentially inhibitors.

## Conclusion

In the present study, we have attempted to search a preferential XOD inhibitor which would discriminately inhibit the 6MP to 6TUA reaction rather than xanthine to uric acid reaction because allopurinol employed in combination with 6MP as cancer chemotherapy inhibits enzymatic hydroxylation of hypoxanthine, xanthine, and 6MP with equal efficiency, which leads to the development of some biochemical problems like xanthinuria [43] and xanthine nephropathy [44]. Furthermore, hypoxanthine when accumulated in the body competes with 6MP for the action by HGPRT [45]. Additionally, it has been observed that the administration of allopurinol, as a component of combination chemotherapy leads to the depletion in the intracellular pool of phosphoribosyl pyrophosphate (PRPP) as allopurinol is converted to allopurinol ribonucleotide by HGPRT using PRPP. The allopurinol ribonucleotide possesses further the potential for inhibition of PRPP amidotransferase and orotidylic acid decarboxylase [45-49]. Conversion to the allopurinol nucleoside by purine nucleoside phosphorylase has also been reported [50].

We found two such preferential inhibitors namely, APT and AHMP. The extent of inhibition is dependent on the binding interactions of the respective substrates and inhibitors with the enzyme.

It is important to mention here that APT is a known anticancer drug with HGPRT as its target enzyme. Therefore, use of APT as a preferential XOD inhibitor along with 6MP may increase the efficiency and reduce the dosage of 6MP. We conclude that administration of either APT or AHMP along with the major anti-leukemic drug 6MP might serve as a good combination cancer chemotherapy regimen. To the extent that adverse consequences of allopurinol therapy may relate to actions of this purine analog compound exclusive of XOD inhibition, the selectivity of AHMP and APT may lessen the risk for at least some of the untoward effects of current combined chemotherapy in patients with cancer. To the best of our knowledge, we have for the first time developed such preferential inhibitors of XOD based on the difference in the interactions with its substrate and inhibitor.

## Methods

AHMP and APT both were purchased from Lancaster, UK. Bovine milk XOD was procured from Calbiochem (La Jolla, CA, USA). Hypoxanthine, xanthine and 6MP were purchased from Sigma (St. Louis, MO, USA). Disodium hydrogen phosphate and Sodium dihydrogen phosphate were procured from Merck Inc. Ltd (Germany). The spectrophotometric experiments were performed on Perkin Elmer's Lambda 25 dual beam spectrophotometer.

XOD activity was determined spectrophotometrically by measuring uric acid formation at 293 nm with xanthine as substrate [51] and 6TUA formation at 350 nm with 6MP as substrate. One enzyme unit is defined as amount of enzyme required to produce 1 μM uric acid per min per ml reaction mixture from 10 μM of xanthine at 30°C, pH 7.4. Equivalent concentration of XOD was found to be 35 nM based on the molar extinction coefficient (Δε) of 36 mM^-1^cm^-1 ^at 450 nm [51]. In all the experiments, the enzyme was added lastly to initiate the reaction. Δε value of uric acid is 11 mM^-1^cm^-1 ^at 293 nm [52] and Δε value of 6TUA is 22 mM^-1^cm^-1 ^at 350 nm.

The IC_50 _values were determined for all the purine and pyrazolopyrimidine-based compounds. Percent inhibition of XOD was studied in terms of decrease in uric acid and 6TUA formation as compared to the product formation in absence of inhibitor. GRAFIT software procured from Erithacus Software Limited, UK, written by Dr R.J. Leatherbarrow, was employed for calculating the IC_50 _values.

To appreciate the actual biochemical interactions carried out by XOD *in vivo *with respect to its natural (xanthine) and synthetic substrates (6MP), we designed a combined competitive *in vitro *simulative experiment wherein we determined the percent residual activity for the formation of uric acid and 6TUA by the co-presence of equimolar concentrations (10 μM, which is higher than the *K*_m _of the two substrates) of both the substrates of XOD (xanthine and 6MP) and either of the two preferential inhibitors (AHMP and APT). Percent residual activity of formation of uric acid and 6TUA was determined by subtracting the percentage inhibition from 100%. *K*_m _of XOD was determined for 6MP and xanthine from the Michaelis Menten plot and the *k*_cat _was determined by using the formula *k*_cat _= *V*_max_/E_t_, where *V*_max _is the maximum velocity attained by a reaction and E_t _is the total enzyme concentration used in the reaction. *K*_m _and *k*_cat _were determined in the absence of the inhibitors. To determine the mechanism of inhibition exhibited, LB plots were drawn using Sigma Plot 9.01 version with Enzyme Kinetics module and the *K*_i _was calculated using the following formulae,

For competitive inhibition,

-1/*K*_m app _= -1/*K*_m _(1+ [I]/*K*_i_)

For non-competitive inhibition,

1/V_max app _= (1+ [I]/*K*_i_)/V_max _

The modified LB equation for the mixed inhibition is

1/v = 1/V_max app _+ *K*_max app_/V_max app _[S]_0 _

where, *K*_m app _and V_max app _are defined by equations (4) and (5).

*K*_m app _= *K*_m _{1+([I_0_]/*K*_i_)}/{1+ ([I_0_]/*K*_I_)}

V_max app _= V_max_/{1+ ([I_0_]/*K*_I_)}

[I_0_] is the total concentration of the inhibitor, [S_0_] is the total concentration of substrate, *K*_m _is the Michaelis constant and V_max _is the maximum velocity of the reaction of XOD with the substrates 6MP or xanthine. *K*_m app _is the apparent Michaelis constant and V_max app _is the apparent maximum velocity of the reaction of XOD with its substrates. *K*_i _and *K*_I _are the inhibition constants of the inhibitors [53,54].

### Evaluation of acute toxicity of two discriminatory inhibitors

In order to evaluate the toxicity of the preferential inhibitors of XOD, the single dose acute toxicity study was performed for both the preferential inhibitors on swiss male mice following a fourteen days observation period. The body weight of the animal ranged from 25–30 gm. Three different doses (2.5, 5, 10 mg/kg) for the two compounds were selected, using allopurinol as the standard drug and administered intravenously [55]. Parameters, e.g., body weight, food intake, mortality, general behavioral toxicity were observed after the administration of the compounds for fourteen days. Gross necropsy of each animal of all groups was conducted and organ weight was recorded after the termination of the study.

## Abbreviations

XOD, Xanthine oxidase; 6MP, 6-Mercaptopurine; 6TUA, 6-thiouric acid; AHMP, 2-amino-6-hydroxy-8-mercaptopurine; APT, 2-amino-6-purinethiol, HGPRT, Hypoxanthine-guanine phosphoribosyl transferase; *K*_i_^1^, Inhibition constant of 2-amino-6-purinethiol; *K*_i_^2^, Competitive inhibition constant of 2-amino-6-hydroxy-8-mercaptopurine; *K*_I_^2^, Non-competitive constant of 2-amino-6-hydroxy-8-mercaptopurine; LB, Lineweaver Burk.

## Authors' contributions

SK carried out the biochemical characterization of the preferential inhibitors by screening the various purine and pyrimidine based compounds and studied the mechanism of the inhibitors. SK assisted in the toxicological studies and also drafted the manuscript. GBJ and KT carried out the toxicological studies of the two preferential inhibitors. AKM conceived the study and participated in its design; principal investigator. All authors read and approved the final manuscript.
